# Usefulness of artificial intelligence in diagnosing early gastric cancer using magnifying endoscopy with narrow-band imaging

**DOI:** 10.1016/j.igie.2025.10.005

**Published:** 2025-10-16

**Authors:** Kenshi Yao, Shunpei Nishida, Masaki Miyaoka, Yoichiro Ono, Takao Kanemitsu, Kentaro Imamura, Satoshi Ishikawa, Rino Hasegawa, Shou Aso, Keisuke Takano, Shugo Ozono, Takayuki Hirase, Takashi Hisabe, Seiichiro Sakaguchi, Manabu Ichikawa, Hidetoshi Nishimura

**Affiliations:** 1 Department of Endoscopy, Fukuoka University Chikushi Hospital, Chikushino, Japan; 2 AI Technology and Solution, Olympus Corp, Hachioji, Japan; 3 Department of Gastroenterology, Fukuoka University Chikushi Hospital, Chikushino, Japan; 4 Advanced Image Processing Technology, Olympus Medical Systems Corp, Hachioji, Japan

## Abstract

**Background and Aims:**

In this study, we developed an artificial intelligence (AI) system to support the diagnosis of early gastric cancer using magnifying endoscopy with narrow-band imaging (NBI), with the goal of improving the diagnostic accuracy of upper gastrointestinal endoscopy. This study aimed to evaluate the diagnostic performance of the AI-based system compared with endoscopists.

**Methods:**

A total of 500 cases, including early gastric cancer and noncancer cases, were used to develop the diagnostic support AI system. We evaluated the AI system with models created using *k*-fold cross-validation. We determined the diagnostic performance of the AI system and 10 endoscopists (5 experts and 5 nonexperts) for detecting early gastric cancer and compared the diagnostic performance between the AI system and endoscopists. Histopathologic diagnosis was used as the reference standard for cancerous lesions, while magnifying endoscopic diagnosis with high confidence served as the reference standard for noncancerous lesions.

**Results:**

The median (interquartile range [IQR]) sensitivity for the AI system and all endoscopists was 100% (100%-100%) and 76.8% (65.5%-78.9%), respectively. The sensitivity of the AI system was significantly higher than that of the endoscopists (*P* = .002). The median (IQR) specificity for the AI system and all endoscopists was 100% (99.0%-100%) and 86.0% (72.0%-91.8%), respectively. The specificity of the AI system was also significantly higher (*P* = .01).

**Conclusions:**

This study demonstrates the feasibility of applying the diagnostic support AI system during magnified observation with NBI and suggests that it could enhance the diagnostic accuracy of upper gastrointestinal endoscopy for early gastric cancer.

## Introduction

In upper gastrointestinal (GI) endoscopy, diagnostic accuracy for early gastric cancer can be improved by magnifying endoscopy with narrow-band imaging (M-NBI) in addition to conventional white-light imaging (C-WLI). A study showed that M-NBI achieved significantly higher accuracy and specificity than C-WLI (90.4% vs 64.8% and 94.3% vs 67.9%, *P* < .001), although sensitivity was not significantly different. When combined with C-WLI, accuracy, sensitivity, and specificity increased to 96.6%, 95.0%, and 96.8%, respectively (*P* < .001).^1^ We developed a vessel-plus-surface (VS) classification system for diagnosing early gastric cancer using M-NBI.^2^^,^^3^ In this system, lesions are assessed for a demarcation line (DL) and for microvascular pattern (MVP) and microsurface pattern (MSP). An irregular MV indicates neovascularization, and an irregular MS indicates epithelial disruption. Lesions with (1) irregular MVP with DL or (2) irregular MSP with DL are diagnosed as cancer; others are noncancerous. This method is widely accepted.^4^

With the complexity of M-NBI, computer-assisted systems are needed. Artificial intelligence (AI) could reduce burden and standardize diagnosis. Previous AI models achieved only mid-80% accuracy, sensitivity, and specificity,^5^ insufficient for clinical use. Thus, an AI system comparable to expert endoscopists is required. This study aimed to investigate the feasibility of such a system.

## Materials and methods

### Study design

This single-center, retrospective study was approved by the Medical Ethics Committee of Fukuoka University Chikushi Hospital, Chikushino, Japan.

### Data acquisition

Consecutive patients who underwent upper GI endoscopy at Fukuoka University Chikushi Hospital (a referral hospital) between January 2014 and September 2022 were considered for inclusion. This study used secondary data collected from patients in a prior study. Endoscopic still images were exported from the Solemio QUEV filing system (Olympus Co, Tokyo, Japan); none of the images contained identifiable personal information. M-NBI still images from 282 patients with early gastric cancer and from 218 individuals without cancer were collected by an expert endoscopist (M.M.) who has experience of more than 1000 M-NBI endoscopy procedures. Endoscopic examinations were performed using upper GI magnifying endoscopes (GIF-Q240Z, GIF-H260Z, and GIF-H290Z; Olympus Co) and EVIS LUCERA ELITE video system (CV-290, Olympus Co).

Images were selected based on the following criteria. Inclusion criteria were (1) magnified endoscopic images captured during procedures performed by expert endoscopists (K.Y., M.M., Y.O., T.K., K.I.), each with experience in more than 1000 magnifying endoscopy cases of the stomach, and (2) images acquired during observation with maximal magnification under narrow-band imaging (NBI). Exclusion criteria included (1) poor-quality images that were out of focus or blurred and (2) images in which assessment of magnified endoscopic findings was impeded by mucus, inflammatory exudate, or bleeding. Initially, the expert endoscopist (M.M.) selected candidate images based on diagnostic relevance. Subsequently, a biomedical engineer (S.N.) independently assessed all images and excluded those with suboptimal quality (eg, blur, mucus, or poor focus) to ensure technical adequacy for training. This sequential process helped maintain both clinical and technical integrity while minimizing selection bias.

A total of 353 M-NBI images from 187 patients with early gastric cancer were used for training, whereas 95 M-NBI images from 95 different patients with early gastric cancer were used for testing. Histopathologic diagnosis was used as the reference standard for cancerous lesions. For noncancer cases, 250 M-NBI images were selected from 118 individuals for training, and 100 M-NBI images from 100 different individuals were used for testing. The diagnoses of these noncancerous lesions included atrophic gastritis, follicular gastritis, erosive gastritis, mucosa-associated lymphoid tissue (MALT) lymphoma, and fundic gland polyps. Biopsies were not performed for noncancerous lesions, except for tumorous lesions such as MALT lymphoma. However, we included lesions that were diagnosed as noncancerous with high confidence based on M-NBI, because high-confidence diagnoses from M-NBI images are comparable to those made from histopathologic analysis of biopsy specimens.^3^

### Annotation

For the extracted images, the VS classification system was used to mark the DL and classify the MVP and the MSP of the lesion (regular, irregular, or absent) (Table 1). All annotations were initially performed by an expert endoscopist (M.M.) with substantial experience in magnifying endoscopy for gastric lesions. A second, more experienced endoscopist (K.Y.) independently reviewed the annotations and made corrections when necessary. This 2-tiered review process ensured consistency in lesion localization and minimized annotation bias.

**Table 1:** Details of findings and diagnoses (early gastric cancer/noncancer) using the VS classification system in the training and test data sets

Endoscopic findings/diagnosis	Training data set (n = 603)	Test data set (n = 195)
Demarcation line (present/absent)	390/213	115/80
Microvascular pattern (regular/irregular/absent)	225/313/65	84/87/24
Microsurface pattern (regular/irregular/absent)	236/303/64	90/83/22
Diagnosis (early gastric cancer/noncancer)	353/250	95/100

*VS*, Vessel-plus-surface.

**Figure 1:**
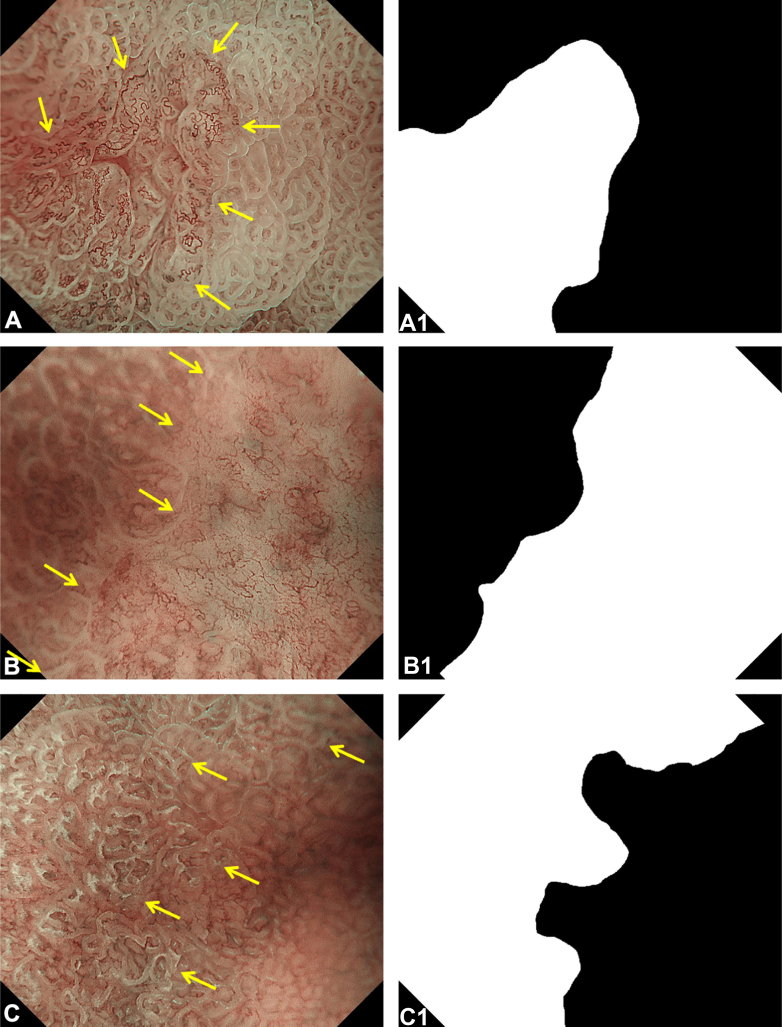

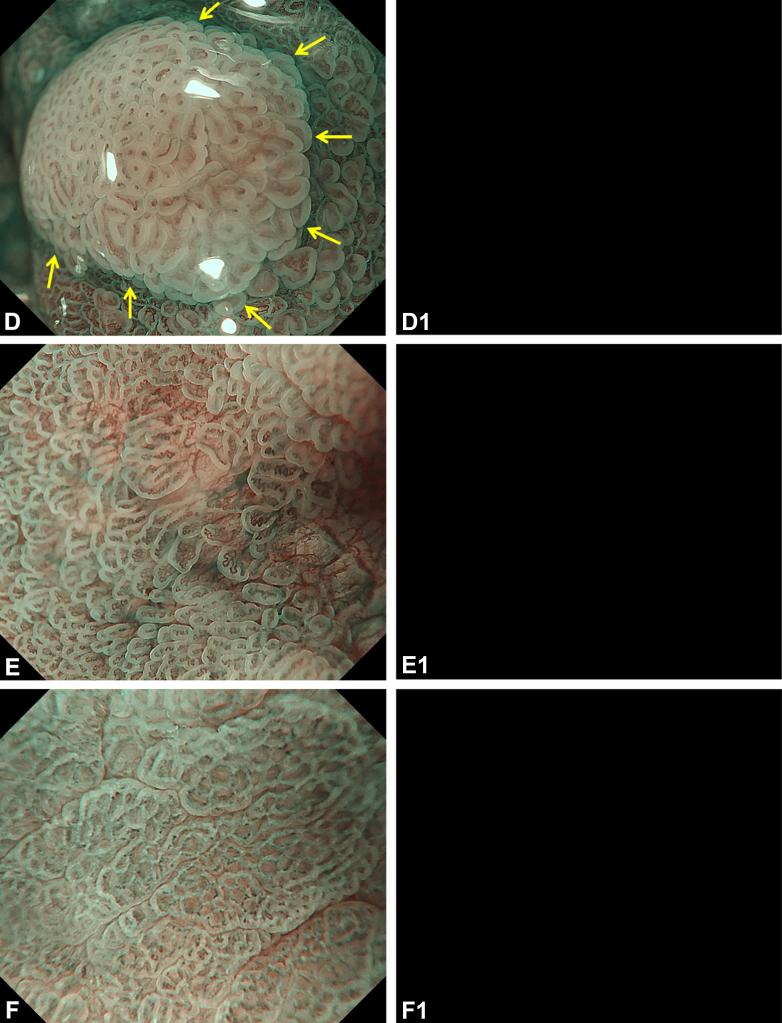
Annotation of M-NBI images: early gastric cancer (**A-C**) and noncancerous lesions (**D-F**). **A,** M-NBI image of early gastric cancer (0-IIa+IIc type). A distinct DL (*arrows*) separates the cancerous mucosa from the noncancerous background mucosa. Within the DL, an irregular MVP and irregular MSP are observed. According to the VS classification system, the lesion is diagnosed as cancer. **A1,** Mask image of panel A, showing the area of early gastric cancer highlighted in white and noncancerous areas in black, based on DL markings. **B,** M-NBI image of early gastric cancer (0-IIc type). A clear DL (*arrows*) is seen between cancerous and noncancerous mucosa. Inside the DL, the mucosa displays an irregular MVP and an absent MSP. The lesion is diagnosed as cancer per the VS classification system. **B1,** Mask image of panel B, showing the cancerous region in white and the remainder in black, based on the DL marking. **C,** M-NBI image of early gastric cancer (0-IIa type). A DL (*arrows*) is visible between cancerous and noncancerous regions. Within the DL, absent MVP and irregular MSP are noted. Diagnosis: cancer. **C1,** Mask image of panel C, showing the cancerous region in white and the noncancerous areas in black. **D,** M-NBI image of a noncancerous hyperplastic polyp. A DL (*arrows*) is present. Both MVP and MSP are regular. Diagnosis: noncancer. **D1,** Mask image of panel D showing all areas in black, indicating noncancerous tissue. **E,** M-NBI image of a noncancerous ulcer scar. DL is absent, with regular MVP and MSP. Diagnosis: noncancer. **E1,** Mask image of panel E showing all areas in black, consistent with noncancerous tissue. **F,** M-NBI image of noncancerous chronic gastritis. DL, MVP are absent; MSP is regular. Diagnosis: noncancer. **F1,** Mask image of panel F showing all areas in black, indicating noncancerous tissue. *0-IIa*, Superficial elevated type; *0-IIa+IIc*, superficial elevated and depressed type; *0-IIc*, superficial depressed type; *DL*, demarcation line; *M-NBI*, magnifying endoscopy with narrow-band imaging; *MSP*, microsurface pattern; *MVP*, microvascular pattern; *VS*, vessel-plus-surface.

To train and evaluate an AI algorithm (semantic segmentation^6^) that differentiates between early gastric cancer and noncancerous areas in endoscopic images, we created a mask image. In this mask, the area of early gastric cancer was highlighted in white, and the other areas, including noncancerous mucosa, were marked in black. Annotation was not performed if it could not be clearly delineated by the DL (Fig. 1).

### Training methods

We selected deep neural networks (DNNs)^7^ suitable for diagnostic support AI systems and performed training using deep learning techniques. The hardware environment for development included a personal computer with an Intel i9-10900X @ 3.70 GHz CPU and an NVIDIA RTX A6000 GPU. The software environment comprised the integrated development environment PyCharm^8^ and the machine learning library PyTorch.^9^

To select the DNN, we used the segmentation_models.pytorch^10^ library, which supports various semantic segmentation algorithms. After testing with a small data set, we identified the combination that yielded the highest accuracy: the DeepLabV3Plus^11^ architecture for the DNN, ResNet101^12^ as the backbone, and ImageNet^13^ for the pretrained model. Initially, we considered using ResNet50, which is lighter than ResNet101. However, because of insufficient model capacity and suboptimal accuracy, we ultimately selected ResNet101.

Data augmentation, a technique used to enhance the quantity and variability of training data through transformations, was applied as a preprocessing step for machine learning.^14^ In this study, we used several data augmentation methods, such as resizing to 512 × 512 pixels, random affine transformations,^15^ horizontal flipping, vertical flipping, and random 90° rotations.

Hyperparameters are external configuration variables used to train machine learning models.^16^ All layers of the convolutional neural network were fine-tuned using stochastic gradient descent^17^ with a global learning rate of 0.00001. The batch size was set to 16, the number of epochs was set to 100, and a sigmoid function was used as the activation function.^18^ The hyperparameters were pretuned and fixed to ensure stable training and validation losses.

*k*-fold cross-validation^19^ is a technique where the training and validation data are divided and recombined multiple times, with training and evaluation performed for each iteration. This method is typically used to check for overfitting in trained AI models. However, in this study, we used *k* = 5, performing 5-fold cross-validation for statistical analysis in the comparison test between the endoscopists and AI.

For training, the population data set (353 M-NBI images of early gastric cancer and 250 M-NBI images of noncancerous tissues) was divided into training and validation data at a ratio of 4:1 to maintain the distribution of the VS classification and meta-annotations. Each subset was then fed into the DNN.

Under these conditions, we input an annotation mask image into the AI model. In this mask, the endoscopic image and region of early gastric cancer were marked in white, whereas all other regions, including noncancerous areas, were marked in black. The model was then trained on these annotated data.

### Evaluation method

**Figure 2:**
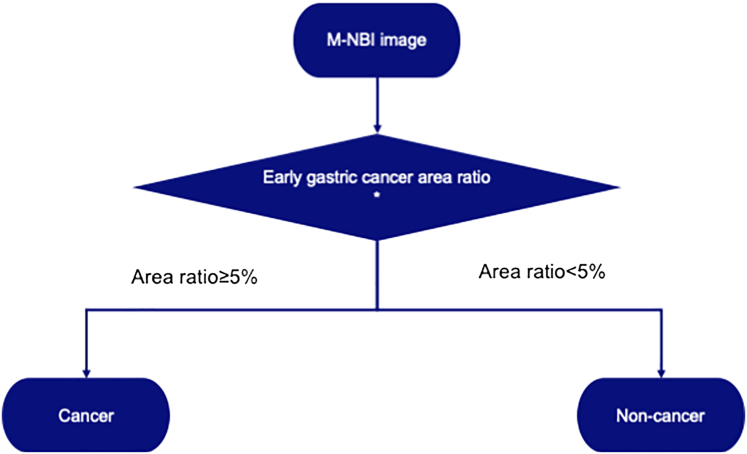
Flowchart of the evaluation method. The early gastric cancer area ratio is defined as the proportion of the endoscopic image, excluding the octagonal mask, classified by the AI algorithm as cancerous. If the AI-determined area ratio is ≥5%, the lesion is diagnosed as cancer. Conversely, if the area ratio is <5%, the lesion is diagnosed as noncancer. Area ratio = (area classified by the AI model as early gastric cancer) / (area of endoscopic image excluding the octagonal mask) × 100%. *AI*, Artificial intelligence; *M-NBI*, magnifying endoscopy with narrow-band imaging.

**Figure 3:**
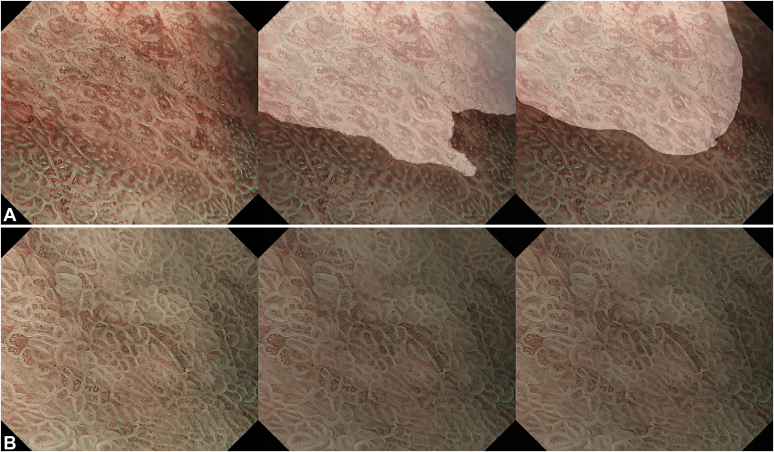
Representative images demonstrating evaluation results classified as true positive and true negative. (Left: original endoscopic image; Center: annotation image; Right: inference result by the AI model). The center image shows the annotation mask created by expert endoscopists, marking regions of early gastric cancer in white. The right image shows the AI model's inference result, with regions predicted as cancer similarly shown in white. Both annotation and inference masks were overlaid onto the original image with 30% transparency to allow visual comparison between actual lesion markings and AI-predicted regions. **A,** Early gastric cancer (true positive), with an area ratio of early gastric cancer ≥5%. **B,** Noncancerous (true negative), with an area ratio of early gastric cancer <5%. *AI*, artificial intelligence.

**Figure 4:**
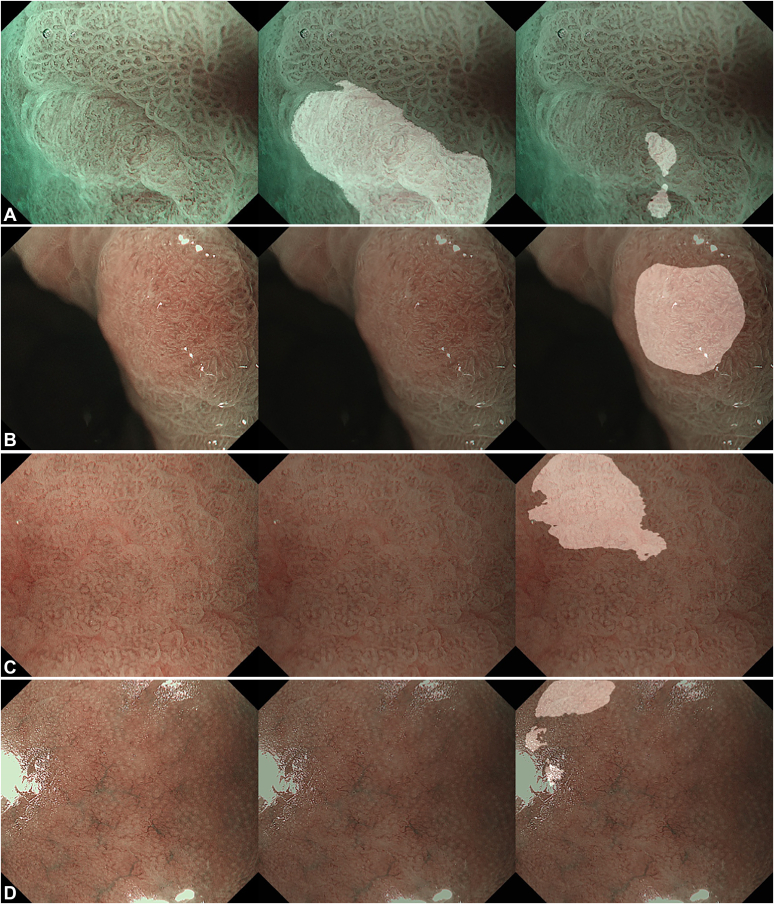
Representative images from patients with false-negative and false-positive evaluation results. (Left: original endoscopic image; Center: annotation image; Right: inference result by the AI model). The annotation images (center) show expert-defined regions of early gastric cancer in white, whereas the inference result images (right) display areas identified by the AI model. Both were overlaid on the original images with 30% transparency for comparison. False-negative cases occurred when the AI failed to detect cancerous regions marked in the annotation (white area with an area ratio of early gastric cancer <5% in the inference image). False-positive cases occurred when the AI incorrectly identified cancer in regions not annotated as malignant (white area with an area ratio of early gastric cancer ≥5% in inference but absent in annotation). **A,** Early gastric cancer: false-negative case. **B,** Noncancerous lesion: false-positive case. **C,** Noncancerous lesion: false-positive case. **D,** Noncancerous lesion: false-positive case. *AI*, Artificial intelligence.

We evaluated the AI model for semantic segmentation by classifying regions in the images as early gastric cancer and noncancerous regions based on the early gastric cancer area ratio of these regions (Figure 2, Figure 3, Figure 4). The area ratio was calculated using the following formula:

Arearatio=(areaclassifiedbytheAImodelasearlygastriccancer)/(areaofendoscopicimageexcludingtheoctagonalmask)×100

This metric quantifies the proportion of the visual field that the AI system classifies as cancerous, providing a spatial indicator of lesion recognition performance. If the area ratio was ≥5%, the region was identified as early gastric cancer. The value ≥5% was set experimentally to remove small detection areas as noise.

### Endoscopist/AI system comparison test method

We compared the diagnostic performance of early gastric cancer between endoscopists and the AI system.

Ten endoscopists (5 experts and 5 nonexperts) from Fukuoka University Chikushi Hospital participated in the study. Endoscopists with ≥500 cases of upper GI magnifying endoscopic observations were classified as experts, whereas those with <500 cases were classified as nonexperts.

The test used 195 endoscopic images (95 with early gastric cancer and 100 noncancer) that were identical to those used to evaluate the AI system. The details of these images were kept confidential from the endoscopists.

The examination was conducted using an internet connection, with a response format distributed to each independent endoscopist, who then answered the questions and returned the completed form. The endoscopists reviewed the images in the format and answered whether the DL, MSP, MVP, and confidence level of the diagnosis were present. All diagnoses (early gastric cancer and noncancer) made by the endoscopists were based on the criteria of the VS classification system. Early gastric cancer or noncancer was automatically inputted based on the DL, MVP, and MSP answers. Additionally, to ensure blinding, the endoscopists were instructed not to discuss the test content with others during the examination.

To evaluate the test, we calculated the specificity, sensitivity, and accuracy of 5 AI models that underwent 5 *k*-fold cross-validation tests with 10 endoscopists (5 experts and 5 nonexperts) and the AI system. We compared the diagnostic performance of the endoscopists and AI system.

### Statistics analysis

The Mann-Whitney *U* test was used to compare the diagnostic performance (specificity, sensitivity, and accuracy) between the 2 groups. Statistical significance was set at *P* <.05. All analyses were performed using EZR software (v1.54; Saitama Medical Center, Jichi Medical University, Tochigi, Japan) and R software (v4.1.1; R Core Team, Vienna, Austria; http://cran.r-project.org/).

## Results

Baseline patient and lesion characteristics for our training and test data sets are shown in Table 2.

**Table 2:** Characteristics of patients with early gastric cancer and the lesions used in the training and test data sets

Characteristics	Training data set (n = 187)	Test data set (n = 95)
Male/female, n	133/54	68/27
Age, years, mean (SD)	74.4 (10.2)	72.7 (9.2)
Tumor location: upper third/middle third/lower third, n	41/94/52	21/41/33
Tumor size, mm, mean (SD)	18.5 (13.0)	18.7 (9.7)
Macroscopic types: 0-I/0-IIa/0-IIb/0-IIc/0 mixed, n	7/40/16/90/34	3/20/5/54/13
Depth of tumor, T1a (mucosa)/T1b (submucosa), n	172/15	85/10

*0-I*, Polypoid type; *0-IIa*, superficial elevated type; *0-IIb*, superficial flat type; *0-IIc*, superficial depressed type; *0 mixed*, superficial elevated plus depressed type/superficial depressed plus flat type; *SD*, standard deviation; *T1a*, tumor confined to the mucosa; *T1b*, tumor confined to the submucosa.

### *k*-fold cross-validation with AI

We performed 5 rounds of *k*-fold cross-validation to divide and recombine the training and validation data, followed by training and evaluation for each AI model. The sensitivity, specificity, and accuracy for the 5 AI models were ≥97%, with no significant bias, indicating high diagnostic performance (Table 3). Among the 5 AI models developed through 5-fold cross-validation (designated AI-1 through AI-5), the specificity, sensitivity, and accuracy of AI-1 and AI-2 were all 100%.

**Table 3:** Diagnostic performance of the AI system and endoscopists for early gastric cancer

Target	Specificity, %	Sensitivity, %	Accuracy, %
AI-1	100.0	100.0	100.0
AI-2	100.0	100.0	100.0
AI-3	99.0	100.0	99.5
AI-4	100.0	98.9	99.5
AI-5	97.0	100.0	98.5
Expert doctor 1	88.0	64.2	76.4
Expert doctor 2	87.0	78.9	83.1
Expert doctor 3	85.0	74.7	80.0
Expert doctor 4	70.0	78.9	74.4
Expert doctor 5	95.0	69.5	82.6
Nonexpert doctor 1	49.0	95.8	71.8
Nonexpert doctor 2	96.0	44.2	70.8
Nonexpert doctor 3	93.0	41.1	67.7
Nonexpert doctor 4	78.0	78.8	78.5
Nonexpert doctor 5	68.0	88.4	77.9

*AI*, Artificial intelligence.

### Comparison of the diagnostic ability of the AI system and endoscopists for early gastric cancer

The diagnostic performances of the AI system and endoscopists for detecting early gastric cancer are shown in Tables 3 and 4, respectively.

**Table 4:** Comparison of diagnostic performance between the AI system and endoscopists for early gastric cancer

Target	n	Specificity, %			Sensitivity, %			Accuracy, %		
		Median	IQR	*P* value∗	Median	IQR	*P* value∗	Median	IQR	*P* value∗
AI	5	100	99.0-100.0		100	100-100		99.5	99.5-100	
All endoscopists	10	86.0	72.0-91.8	.002†	76.8	65.5-78.9	.002‡	77.2	72.5-79.6	.002†
Expert endoscopists	5	87.0	85.0-88.0	.011#	74.7	69.5-78.9	.011‡	80.0	76.4-82.6	.009#
Nonexpert endoscopists	5	78.0	68.0-93.0	.011∗∗	78.9	44.2-88.4	.011‡	71.8	70.8-77.9	.009∗∗

*P* values represent comparisons of diagnostic performance metrics (sensitivity, specificity, and accuracy) between the AI system and endoscopists.

*AI*, Artificial intelligence; *IQR*, interquartile range.

∗ Mann-Whitney *U* test.

† AI versus all endoscopists.

‡ Statistical significance was set at *P* < .05.

# AI versus expert endoscopists.

∗∗ AI versus nonexpert endoscopists.

The median sensitivity (interquartile range [IQR]) of the AI system for diagnosing early gastric cancer was 100% (100%-100%). In contrast, the median sensitivity (IQR) of all endoscopists was 76.8% (65.5%-78.9%). The sensitivity of the AI system was significantly higher than that of all the endoscopists (*P* = .002). The median sensitivity (IQR) for expert doctors was 74.7% (69.5%-78.9%), and for nonexpert doctors, it was 78.9% (44.2%-88.4%). The sensitivity of the AI system was significantly higher than that of expert and nonexpert doctors (*P* = .011 and *P* = .011, respectively).

The median specificity (IQR) of the AI system in diagnosing early gastric cancer was 100% (99.0%-100%). In comparison, the median specificity (IQR) for all endoscopists was 86% (72.0%-91.8%). The specificity of the AI system was significantly higher than that of all the endoscopists (*P* = .002). The median specificity (IQR) for expert doctors was 87.0% (85.0%-88.0%), and for nonexpert doctors, it was 78.0% (68.0%-93.0%). The specificity of the AI system was significantly higher than that of both expert and nonexpert doctors (*P* = .011 and *P* = .011, respectively).

The median accuracy (IQR) of the AI system for diagnosing early gastric cancer was 99.5% (99.5%-100%). In contrast, the median accuracy (IQR) of all endoscopists was 77.2% (72.5%-79.6%). The accuracy of the AI system was significantly higher than that of all the endoscopists (*P* = .002). The median accuracy (IQR) for only expert doctors was 80.0% (76.4-82.6%), and for nonexpert doctors it was 71.8% (70.8%-77.9%). The accuracy of the AI system was significantly higher than that of both expert and nonexpert doctors (*P* = .009 and *P* = .009, respectively).

The median sensitivity (IQR) for expert endoscopists was 74.7% (69.5%-78.9%), and for nonexpert endoscopists it was 78.9% (44.2%-88.4%), with no statistically significant difference (*P* = .75). The median specificity (IQR) was 87.0% (85.0%-88.0%) for experts and 78% (68.0%-93.0%) for nonexperts (*P* = .69). The median accuracy (IQR) was 80.0% (76.4%-82.6%) for expert endoscopists and 71.8% (70.8%-77.9%) for nonexperts (*P* = .10). These findings indicate that there was no significant performance difference between expert and nonexpert readers in this data set.

## Discussion

The AI system developed in this study demonstrated exceptionally high diagnostic performance in differentiating between cancerous and noncancerous lesions using M-NBI images. Its diagnostic performance was superior to that of endoscopists, with a statistically significant difference. Although previous studies have compared the diagnostic performance of endoscopists and AI,^5^ showing a sensitivity of 87.4%, specificity of 82.8%, and accuracy of 85.1%, it remains difficult to conclude that this level of performance is sufficient for clinical use.^3^ Other studies have reported AIs with diagnostic performance similar to that of this study, but they have not compared AI performance to that of endoscopists.^20^ Furthermore, Ueyama et al^20^ used M-NBI images captured using the water immersion technique. In contrast, the M-NBI images in the current study were not obtained under special conditions, making the results more clinically reproducible and universally applicable than those of the previous report.^20^

A comparison of the diagnostic performance of AI and endoscopists for early gastric cancer showed that the sensitivity, specificity, and accuracy of AI were significantly higher than those of both expert and nonexpert endoscopists. Based on these results, we explored why AI achieved higher sensitivity, specificity, and accuracy than endoscopists.

To build a high-precision AI system, 4 key requirements are critical: problem setting, large amounts of data, high-quality data, and appropriate learning methods, all of which contribute to model performance. Regarding the problem setting, we adopted a segmentation AI system that leveraged the characteristics of M-NBI to capture the boundary between early gastric cancer and noncancerous areas. This approach allowed us to develop a system that classified early gastric cancer and noncancerous areas into binary categories based on the area ratio of the images.

For the large amount of data, we collected 353 training images and 95 evaluation images for early gastric cancer and 250 training images and 100 evaluation images for noncancerous lesions. Although the absolute amount of data was not large, high accuracy was achieved through generalization within a single facility. For high-quality data, DL, MVP, MSP, and diagnostic confidence were assigned by expert doctors, resulting in high-quality data. Based on the VS classification, the issues and countermeasures were organized, which led to high accuracy by specifically addressing images that were challenging to learn.

For the learning method and model performance, we tuned the architecture, backbone, and hyperparameters at the minimum data stage to build a robust model. We believe that by meeting these requirements, we can achieve high accuracy.

**Figure 5:**
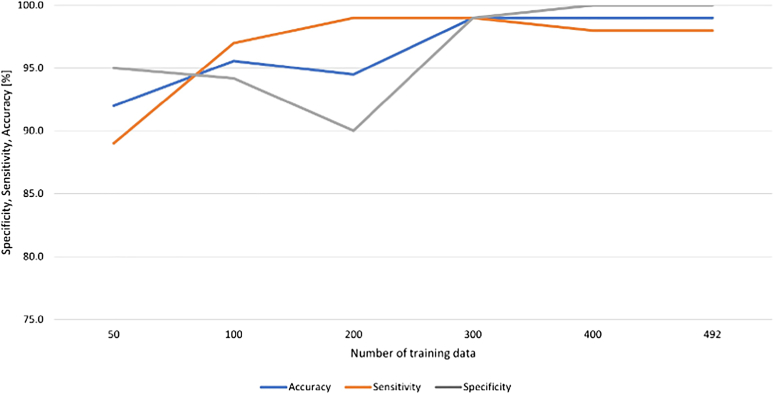
Learning curves of diagnostic performance for early gastric cancer (positive) versus noncancer (negative). Specificity, sensitivity, and accuracy were calculated for the binary classification of early gastric cancer (positive) and noncancer (negative). To assess whether performance improvements were consistent and not driven by an arbitrarily selected training sample size, we intentionally varied only the number of training samples while keeping the evaluation (test) data set fixed. Each model was trained using the same program and training settings as those used for AI-1 through AI-5 and then evaluated on the fixed test data set. *AI*, Artificial intelligence.

Next, we verified that the results were correct and accurate and not due to human error. We investigated whether images in the training and validation data were identical or extremely similar to the evaluation data, and no such errors were observed. We also investigated the presence of any irregularities in the learning curve. To this end, we generated multiple data sets by varying the number of training samples and trained each data set using the same program applied to AI-1 through AI-5. Each resulting model was then evaluated against the corresponding evaluation data set for AI-1 through AI-5. We found that the specificity, sensitivity, and accuracy for discriminating early gastric cancer (positive) from noncancer (negative) increased as the amount of training data increased, while the evaluation data set was kept fixed, and no anomalous or nonmonotonic trends were observed (Fig. 5).

Additionally, to check whether the training and validation data were overfitted to the evaluation data, *k*-fold cross-validation was conducted by dividing and recombining the training and validation data. The results confirmed that the overall accuracy was high, and no abnormalities were found. The probability of a positive assessment for each pixel was displayed as a heat map, ranging from 0% to 100%, and an investigation was conducted to determine whether only the early gastric cancer region could be correctly assessed with a high probability. No abnormalities were found.

The threshold for a positive result was set at 90%; however, we also checked the positive and negative results when the threshold was lowered to 80% and 70%, respectively, to investigate the robustness of the threshold. All questions were answered correctly for all thresholds, and no abnormalities were found.

Based on the above 2 verifications, it was suggested that the evaluation data set was at least accurate and highly reliable. The 5 models in the *k*-fold cross-validation demonstrated very high diagnostic accuracy; however, there were some misdiagnosed cases. We reviewed these cases and analyzed the factors that led to the misclassifications in the evaluation.

First, we described cases in which early gastric cancer was misclassified as noncancerous (false-negative cases). As shown in Figure 4A, there were cases in which the early gastric cancer regions with irregular MVP and irregular MSP could not be identified because indigo carmine was sprayed. We suspect that this was due to the limited amount of data available for learning in cases where indigo carmine was used.

Next, we described cases in which noncancerous conditions were misclassified as early gastric cancer (false-positive cases). As shown in Figure 4B and C, there were cases in which the texture of the fine vascular structure was incorrectly assessed as an early gastric cancer region. We suspect that this occurred because the training data for early gastric cancer included many fine vascular structures of irregular MVP, which could lead to misinterpretation of the MV pattern.

Additionally, there were cases in which nonatrophic gastric mucosa was mistakenly assessed as early gastric cancer (Fig. 4D). In this study, we hypothesized that the cause was the limited amount of learning data for nonatrophic mucosa, which closely resembled normal tissue, with little progression of gastritis.

The following limitations of this study and further studies are essential for the future. First, the outcomes were based on the data using not video clips but still images. The final design of the AI is based on still images obtained when the endoscopist captured these images during the procedure. Accordingly, the situation using the still images seems to be quite similar to that of the original design. Because this was not an on-site study (ie, it was not conducted in a clinical setting), we are planning a prospective feasibility study to test this AI in real clinical practice in the near future. Second, this study was conducted at a single center, and the number of images in the test data set is small. As a result, the model's diagnostic performance may reflect institutional practices or patient selection biases specific to our setting, which could limit generalizability to other clinical environments. To address this, we plan to conduct multicenter prospective studies with a larger and more diverse image data set. Third, only clear, high-magnification M-NBI images were used. Fourth, gastric adenocarcinoma of the fundic gland type and diffuse-type early gastric cancer were excluded from the learning and evaluation targets, as they present diagnostic limitations within the VS classification system. Fifth, this study used images obtained from earlier-generation magnifying endoscopes. Because more advanced endoscopic systems with improved resolution and imaging algorithms have been introduced after this study, further validation is necessary to determine how the AI model performs with these newer platforms. Additionally, cross-platform validation including systems from different manufacturers is needed to assess its robustness across imaging environments. Sixth, we used high-confidence endoscopic diagnosis by M-NBI as a reference standard for noncancerous lesions because we have already demonstrated that high-confidence optical diagnosis by M-NBI closely approximates histopathologic findings in screening endoscopy for early gastric cancer. This limitation could influence model performance.

In conclusion, this study suggests the feasibility of an AI system that could support the diagnosis of early gastric cancer using M-NBI. The sensitivity, specificity, and accuracy of the AI system were significantly higher than those of endoscopists, including expert doctors.

These results represent an important step toward the development of practical AI that can contribute to improving the accuracy of diagnosis in upper GI endoscopy. Further studies and improvements are needed for practical applications in clinical settings.

## Patient consent

This study used secondary data collected from patients in a prior study after obtaining written informed consent. The consent form included provisions for the use of these data in subsequent related research.

## Disclosure

The following authors disclosed financial relationships: S. Nishida, S. Sakaguchi, and H. Nishimura: Employees of Olympus Corporation. M. Ichikawa: Employee of Olympus Medical Systems Corporation, a subsidiary of Olympus Corporation. All 4 authors receive their salaries from Olympus Corporation. All other authors disclosed no financial relationships.

Fukuoka University Chikushi Hospital and Olympus (including Olympus Corporation and Olympus Medical Systems Corporation) have a joint research agreement. There were no financial transactions between the 2 entities in relation to this study. Olympus contributed to the study concept, design, methodology, AI development, and evaluation, but was not involved in data acquisition.
